# Supplementary material for: On the forms, contributions and impacts of community mobilisation involved with Kerala’s COVID-19 response: Perspectives of health staff, Local Self Government institution and community leaders
Source: PLoS One. 2023 Jun 6;18(6):e0285999. doi: 10.1371/journal.pone.0285999 (PMC10243625; doi:10.1371/journal.pone.0285999)
Supplement: S1 File — (DOCX) [file pone.0285999.s001.docx]

**Description of the dataset**

1. Aarogya sena was started 5 years ago. In a locality of 25 houses, somebody who stays there was assigned,… If some disease happens there or some communicable diseases occur there, they will come to know... At that time (pre-COVID time), this did not work out well. But once COVID came, several people started volunteering. Now we have a really good RRT team...They take the initiative for several issues. They work really well as informers and for source reduction (communicable disease control). (41_MO)
2. RRT was formed initially as a disaster management group during the floods. We then diverted the group to COVID response. We could mobilise the RRT as a response to both these crisis, even though the ways to handle a flood and pandemic are completely different.(41_CL)
3. I chose 22 members for the RRT [in our ward]. This is more than the number they had suggested. Among them, 18 are men and 4 are women. By women, I mean girls. To [supervise] this group, we also had two teachers… They are government employees. They helped us. We publicised four contact numbers as the control room. One was mine, one belonged to one of these teachers, my wife's number and another woman's number. (32_CL)
4. …during the first phase of COVID, before any others in the (anonymised) district, we met at the Panchayat level... we selected 10 volunteers from each ward and prepared a list of almost 160 volunteers. We selected a leader from each ward...So we had a working system earlier and later it became a common trend, and it was made official. We had formed the RRT by then…There was training at the Panchayat level through Zoom. (11_MO)
5. The training they received as per the government instruction made them able to understand the situation at the Panchayat level and act accordingly. (22_CL)
6. We (Panchayat) bought masks, sanitisers and other peripherals for the volunteers (RRTs) to fulfil their duties. (22_PP)
7. If someone's condition worsens and there is an emergency, they [the RRTs] provide ambulance service…They deliver food (and medicines) for patients. Sometimes all the medicines would not be available in our pharmacy. They get it from other pharmacies as soon as I call and inform them and deliver it to the patients. (31_HSC)
8. One incident that happened in my ward was the sudden death of a person due to COVID. He only had a brother and unfortunately, none of the family members were able to claim the dead body due to risk of infection. Members of (political party – name masked) were ready to carry his body wearing PPE [Personal Protective Equipment] kits at the time. (41_PP)
9. Many gave access to their buildings to shelter COVID patients…, sponsored different things…In their respective wards, ward members unified these efforts. With the cooperation from many departments, these activities were done very well. Similarly, many volunteers came forward to manage crowds [at the COVID vaccination centres and general crowd as well] and ensure [the] COVID protocol was followed. (42_MO)
10. In ward level we have Jagratha Samithi (health force led by health department and LSG)… The convener of the Jagratha Samithi is an RRT member and the chairman is the ward member. Then we have a team of people which is only in the (masked the name) district, named as MASH. Other than them, we have 3 or 4 local people and police. So, this is the team known as Jagratha Samithi. Every ward has this kind of team, and they are having regular meetings and work. But I think it should be more efficient. (12_MO)
11. We used to manage all those roles alone (COVID control activities) but with the introduction of RRT and social involvement, our burden was reduced substantially…So our roles were reduced because of the social responsibility initiative. One benefit because of the social responsibility initiative was that it made people aware of their roles and responsibilities... Now it is possible to tackle any other disease with this structure. That is the benefit. (22_JHI)
12. Especially during COVID, they [RRTs] have been useful. Prior to the pandemic, such groups have been mostly inactive…But during the crisis we are able to utilise them in several ways…We deliver it [medicines] for COVID patients, their families as well as to those in the primary contact list [and to] the NCD patients as well. We do this with the help of our volunteers. Apart from the institution staff, we have so many volunteers including students. (31_MO)
13. RRT was formed during COVID time. …since it was lockdown and people couldn't…go for their jobs, they were able to spend a lot of time in voluntary services…Later when the lockdown was lifted, people went back to their jobs. (11_HI)
