# Supplementary material for: On the forms, contributions and impacts of community mobilisation involved with Kerala’s COVID-19 response: Perspectives of health staff, Local Self Government institution and community leaders
Source: PLoS One. 2023 Jun 6;18(6):e0285999. doi: 10.1371/journal.pone.0285999 (PMC10243625; doi:10.1371/journal.pone.0285999)
Supplement: S2 File — (DOCX) [file pone.0285999.s002.docx]

**Interview Topic Guide**

1. Recent health reforms
2. What according to you were the major health reforms in Kerala in the last five years? Why do you consider them major? (Probe: Aardram, if not mentioned)
3. (How) Were you involved in these?
4. What were the guiding principles of these reforms?
5. What was prioritized?
6. Which populations were prioritized?

1. Governance and management
2. How was the process of the reforms you mentioned arrived at? Who was involved? Who was not?
3. What was the nature of training and guidelines for program implementation?
4. Describe the administrative challenges if any, faced during implementation of program (Probe: human resource, funds)

1. Roles and responsibilities
2. Please describe the role of Local self-government in these recent health reforms? In health delivery?
3. What has been the role of communities in health reforms? In health delivery? (Probe:   “Arogya sena” or voluntary support, other community groups or formations)
   1. What lessons do you think these experiences offer? Please give examples

1. Impact of health reforms
2. Taking the example of the reforms you just mentioned, what do you believe has been the impact on Kerala’s people? Please give examples
3. What according to you were missing in the scope of health reforms in the past?

1. Impact of COVID 19 in health care delivery last year
2. What were the major duties you were involved in in relation to the COVID 19 pandemic?
3. Please describe how COVID-19 affected your work in health. Please give examples
4. What about groups that are already hard to reach or face exclusion? How were they affected?
5. How were such groups reached? Please give examples

1. Heath care for excluded populations
2. In your view, were these groups already facing exclusion?
   1. If no, (what) did COVID introduce in terms of new vulnerabilities/challenges
   2. If yes, what kinds of exclusion/challenges were these groups already facing?
3. Do special/unique strategies exist for these groups? Please give specific examples
4. What about groups within groups – were there population groups within advantaged or even disadvantaged groups for whom where services, coverage or institutions would be less than other groups?
   1. Probe: women, younger or older age-groups, certain occupational groups (including migrants), SC low income
   2. Probe: based on our data <report group> appears to face exclusion in accessing chronic care. What has been your experience in providing care with this group?

1. If you were to make a list of 3 to 5 strategies to improve inclusivity of health reforms – to make sure no one would be left behind, what would you suggest? (probe: who is responsible for each strategy?)
