# Supplementary material for: On the forms, contributions and impacts of community mobilisation involved with Kerala’s COVID-19 response: Perspectives of health staff, Local Self Government institution and community leaders
Source: PLoS One. 2023 Jun 6;18(6):e0285999. doi: 10.1371/journal.pone.0285999 (PMC10243625; doi:10.1371/journal.pone.0285999)
Supplement: Attachment — Submitted filename: Kerala COVID Comm Mob_Response to Reviewers.docx [file pone.0285999.s003.docx]

**Response to Reviewers and Editor**

**Comments to the Author**

1. Is the manuscript technically sound, and do the data support the conclusions?

The manuscript must describe a technically sound piece of scientific research with data that supports the conclusions. Experiments must have been conducted rigorously, with appropriate controls, replication, and sample sizes. The conclusions must be drawn appropriately based on the data presented.

Reviewer #1: Yes

Reviewer #2: Yes

Thank you for reviewing the manuscript.

2. Has the statistical analysis been performed appropriately and rigorously?

Reviewer #1: N/A

Reviewer #2: N/A

Thank you for reviewing the manuscript.

3. Have the authors made all data underlying the findings in their manuscript fully available?

The [PLOS Data policy](http://www.plosone.org/static/policies.action#sharing) requires authors to make all data underlying the findings described in their manuscript fully available without restriction, with rare exception (please refer to the Data Availability Statement in the manuscript PDF file). The data should be provided as part of the manuscript or its supporting information, or deposited to a public repository. For example, in addition to summary statistics, the data points behind means, medians and variance measures should be available. If there are restrictions on publicly sharing data—e.g. participant privacy or use of data from a third party—those must be specified.

Reviewer #1: Yes

Reviewer #2: No

The reviewer is correct. We have revised the statement to the following *“**The datasets generated during and/or analysed during the current study are available from the corresponding author on reasonable request.*” in the supplementary file.

4. Is the manuscript presented in an intelligible fashion and written in standard English?

PLOS ONE does not copyedit accepted manuscripts, so the language in submitted articles must be clear, correct, and unambiguous. Any typographical or grammatical errors should be corrected at revision, so please note any specific errors here.

Reviewer #1: Yes

Reviewer #2: Yes

Thank you for reviewing our manuscript.

5. Review Comments to the Author

Reviewer #1: In this study, the authors investigate the role of community participation in the response to the COVID-19 pandemic in Kerala, India, using interviews and thematic analysis. Results highlighted the formation and contributions of Rapid Response Teams (RRT). The manuscript is an interesting exploration of the interactions between volunteer groups and government-run public health. Questions and comments are below:

1. Background- aside from providing PPE, was any government funding or other resources provided to community groups working in the COVID-19 response? What was the source of this funding?

Volunteers had received only PPE in material form. This is already indicated in line 271 and we have indicated the funding source in the discussion section in line 431.

2. I was not able to access the interview questions as they were not included in the supplementary file. Were these questions piloted prior to starting interviewing and data collection?

The interview quide has been added to the supplementary file in this round of revision and thanks for pointing this out. Also, in line 161 we have already mentioned that the tool was piloted.

3. How did the role of the RRTs evolve over the course of the pandemic?

Through the course of results section, we have discussed the different roles that RRT had been assigned with and how they were entrusted with different roles as the requirements were changing. While patient transport, food and medicine delivery, assisting in funerary arrangements etc. were the initial responsibilities, helping people to register for COVID vaccination through digital media was a role that emerged later.

4. How did the pandemic response and the role of community groups in Kerala compare to other states in India?

In line 421-423, we have mentioned about the presence of similar informal collectives and civil society mobilizations for pandemic response in other states in India. However, the difference was that there existed a disconnect between people and health systems which Kerala was trying to solve using community volunteers.

5. Most of the results and discussions focus on the beneficial role that the RRTs played, which is an important finding. However, I am interested in what respondents had to say about challenges and problems they experienced. Could these results be added?

Thanks for this question. We went through our data again to check if anything we can draw on this aspect. However, no participant has pointed to challenges they faced working with RRTs.

Minor comment: Please write out all abbreviations upon first use (e.g. ASHA. LSGD) as many of these will not be familiar to readers.

The suggestion is well received. We have done another round of proof reading and added expansions wherever it was missed.

Reviewer #2: Dear Editor,
I really appreciate the opportunity to review the manuscript PONE-D-22-29723 entitled:
"On the forms, contributions and impacts of community mobilisation involved with Kerala’s COVID-19 response: Perspectives of health staff, local self government institution and community leaders"

I commend the authors for describing this critical and timely issue. The paper is interesting and well-written; however, I would like to highlight some issues that merit revision:

The text appears very well written and interesting, but in some cases there are obvious typos or unclearly written sentences, such as, "and development and are trained in." In this regard, I ask the authors to carefully review the text of the manuscript.

Thanks for flagging it. We have done another round of proof reading to correct the misspelt words and to improve unclear sentences.

I did not find several acronyms written in full, e.g. LSGD (LSG is found, but not LSGD), and so also
"HFWD";; I guess it is the same as DHFW, but in the text the acronyms should be clear and unicvoche for the reader, please, I ask the authors to evaluate and correct quesot aspetot.

Thanks for pointing this out. We have done a thorough proof reading and added expansions wherever it was missed.

Finally, in the conclusion, I found overenthusiastic tones. I fully understand that this is a good thing but writing "COVID-19 pandemic created opportunities for community participation to expand" exposes the paper to misinterpretation by uncaring journalists, who might titrate something like "covid brought good things." I ask the authors to mitigate the sentence with a small initial addition such as "although it brought harm to society and victims..."

This is a great point. We have added more content about community only being given “invited space” which does not include policy planning etc. This is also reflected in the conclusion (lines 486-495).

6. PLOS authors have the option to publish the peer review history of their article ([what does this mean?](https://journals.plos.org/plosone/s/editorial-and-peer-review-process#loc-peer-review-history)). If published, this will include your full peer review and any attached files.

If you choose “no”, your identity will remain anonymous but your review may still be made public.

**Do you want your identity to be public for this peer review?** For information about this choice, including consent withdrawal, please see our [Privacy Policy](https://www.plos.org/privacy-policy).

Reviewer #1: No
Reviewer #2: **Yes:** Massimo Tusconi
